# Process Mapping of the Sol–Gel Transition in Acid-Initiated Sodium Silicate Solutions

**DOI:** 10.3390/gels10100673

**Published:** 2024-10-21

**Authors:** Marzieh Matinfar, John A. Nychka

**Affiliations:** Department of Chemical and Materials Engineering, University of Alberta, Edmonton, AB T6G 1H9, Canada; jnychka@ualberta.ca

**Keywords:** silica gel, colloidal silica, sodium silicate solution, sol–gel, gelation kinetics, optical properties, bone tissue scaffold

## Abstract

Fabricating large-scale porous bioactive glass bone scaffolds presents significant challenges. This study aims to develop formable, in situ setting scaffolds with a practical gelation time of about 10 min by mixing 45S5 bioactive glass with sodium silicate (waterglass) and an acid initiator. The effects of pH (2–11), waterglass concentration (15–50 wt.%), and acid initiator type (phosphoric or boric acid) were examined to optimize gelation kinetics and microstructure. A 10 min gelation time was achieved with boric acid and phosphoric acid at various pH levels by adjusting the waterglass concentration. Exponential and polynomial models were proposed to predict gelation times in basic and acidic environments, respectively. The optical properties of the gels were studied qualitatively and quantitatively, providing insights into gelation kinetics and structure. Acidic gels formed smaller particles in a dense network (pores < 550 nm) with higher light transmittance, while basic gels had larger aggregates (pores ~5 µm) and lower transmittance. As the waterglass concentration decreased, pore size and transmittance converged in both groups. The onset of gelation was detected around 8 min using the derivative of light transmittance. This work identifies the key factors controlling waterglass gelation and their impact on gel structure, enabling the tailored creation of formable, in situ setting bioactive glass bone scaffolds.

## 1. Introduction

The very first material that was found to form a bond with bone was the original bioactive glass composition, 45S5 Bioglass (45 wt% SiO_2_, 24.5 wt% CaO, 24.5 wt% Na_2_O, and 6 wt% P_2_O_5_), which has been in clinical use since 1985 [1]. Since then, particles and putties containing a variety of bioactive glass particulates have been widely used in clinical settings. However, surgeons sometimes require large interconnected macroporous scaffolds for the regeneration of large bone defects. These 3D porous structures, known as scaffolds, act as temporary templates to support and stimulate bone regeneration while they gradually degrade and are eventually replaced by new bone tissue.

The first attempt to produce a bioactive glass-based scaffold was made in 2002 using a sol–gel process combined with in situ foaming to obtain a macroporous structure [2]. Since then, many researchers have worked to identify the optimal manufacturing process for fabricating an “ideal” scaffold from bioactive glass. Multiple attempts have been made to define what constitutes an ideal scaffold [3,4,5], which can be summarized by the following points:

- Sufficient mechanical properties and controllable degradation rate, capable of providing short-term strength that transitions into load-bearing bone before being resorbed;
- Ability to bond to bone (bioactivity);
- Interconnected porosity and controllable pore size to allow for cell growth and vascularization;
- Reliable, repeatable, fast, and economically convenient for mass production;
- Free from any toxic substances and safe for people and the environment;
- Customizable to meet the needs of each patient.

**Figure 1:**
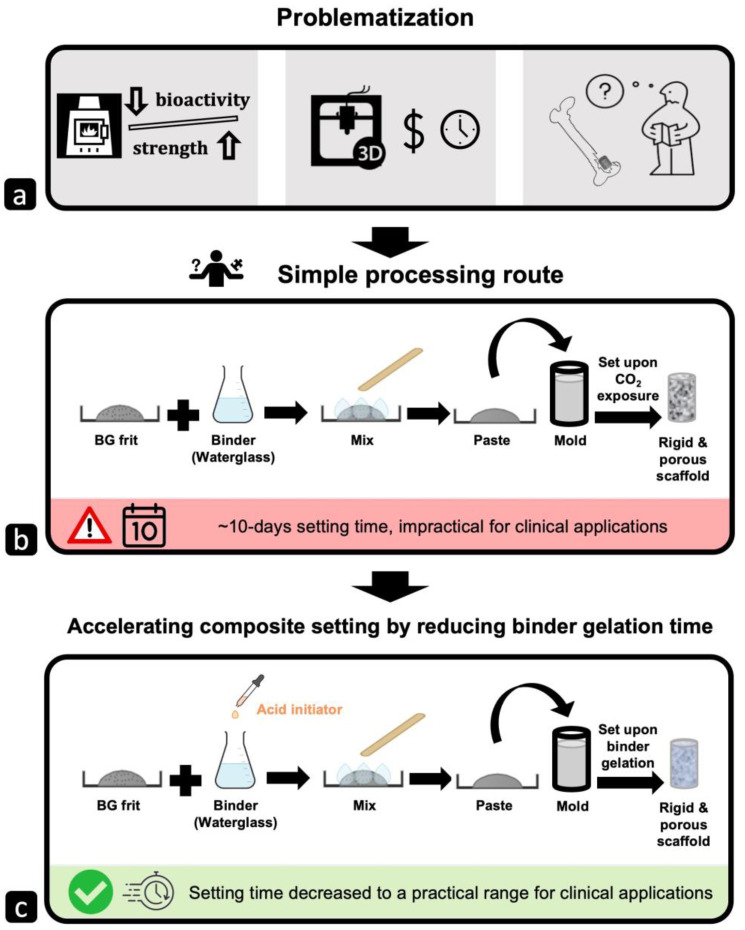
Progression from problematization to design approach in this study: (**a**) three major challenges were spotted applying problematization in bioactive glass bone scaffold processing techniques; (**b**) to address the problems in section (**a**), a new processing technique (the previous proof-of-concept work) was developed which resulted in bioactive glass composites bone scaffolds with long setting time impractical for clinal application; (**c**) developing a new approach using acid-initiated waterglass to reduce the setting time to a practical range for clinical applications.

The technologies developed so far for producing glass-based scaffolds can be categorized into two main groups: (1) conventional methods and (2) additive manufacturing techniques (AMTs), also known as rapid prototyping (RP). The first group follows the top-down manufacturing approach, which involves removing selected pieces or parts from a bulk material to create the desired shape and porosity. The second group uses a bottom-up approach, building the scaffold layer-by-layer or piece-by-piece. Details about the methods in each group and their advantages and disadvantages can be found elsewhere [4,6]. Despite the variety of techniques available, there are still no large-scale porous bioactive glasses on the market. Applying problematization [7] on the field of processing methods for ceramic-based tissue scaffolds revealed three major challenges (Figure 1a):

1. Conventional methods require heat treatment for consolidation, which adversely affects glass bioactivity;
2. AMTs, such as 3D printing, require 3D models and imaging for each patient, which can be costly and time-consuming;
3. There is a lack of formability, despite bone defects coming in various sizes and shapes.

Suppose we could create a binary mixture of a powder and a liquid binder that a surgeon can form into a paste and then press directly into a bone defect in a patient, and let the implant set (i.e., harden to prevent migration from the wound site) in seconds to minutes. As a proof-of-concept, a design was explored in our lab in which 45S5 glass powder was combined with a sodium silicate solution binder to create a formable paste that sets upon exposure to CO_2_ gas in the air and hardens into a rigid, porous scaffold [8,9]. Sodium silicate solution, also known as waterglass, was chosen as a binder phase for its ability to set at room temperature, efficiently wet and coat the glass surface, and set glass frit into a porous 3D structure. Additionally, some reports indicate the promise of sodium silicates for biomedical applications. For example, soluble silicates were used in the fabrication of dense calcium phosphate-based bone cement [10,11,12] and drug delivery agents [13]. In these examples, sodium silicate solution not only served as a hardening liquid but also controlled various properties of the composites, including setting time, pH, in vitro degradation rate, and mechanical properties. Even more promising, silica gel coatings on polymeric substrates were shown to biomineralize when immersed in simulated body fluid [14,15]. Therefore, waterglass has the potential to be used as a “bioactive binder”.

The proof-of-concept studies [8,9] showed that fabrication of porous bioactive glass composites at ambient temperature is possible: a formable paste was successfully made by combining waterglass and bioactive glass that set in the air with geometric stability, and the resulting structure of the compacts was porous with sufficient compressive strength to permit handling. However, the setting time of these bioactive glass composite bone scaffolds were more than 10 days (~300 h), making them impractical for clinical use (Figure 1b). Therefore, the main goal of this research is to decrease the setting time of bioactive glass–waterglass composite bone scaffolds to a range that is practical for clinical applications. Specifically, we aim to develop a workable, formable paste that can be inserted inside a bone defect of any size and shape that can be set in situ into a rigid, porous 3D structure, promoting bone tissue regrowth in a safe and effective manner.

To decrease the binder setting time, we first need to understand the setting mechanism. Sodium silicate solutions, with the general formula of (Na_2_O)_x_·(SiO_2_)_y_·(H_2_O)_z_ (x, y, z = molar ratio), are complex mixtures of water, anionic silicate species, and sodium cations, in dynamic equilibrium. The SiO_2_ concentration and the SiO_2_:Na_2_O ratio are the two main factors that govern the physical and chemical properties of these solutions. Waterglass can readily transform into silica gel upon acidification. Silica gel formation is initiated by destabilizing waterglass with acids (HX), which triggers the partial neutralization of Si–O^−^ Na^+^ ion pairs and leads to the formation of reactive silanol (Si–OH) groups and sodium salts [16] (Equation (1)). Silanol groups on the surface of silicate particles connect by forming Si–O–Si bridges or physical interactions, forming silicate aggregates that form a 3D network of silica gel. Both the formation of reactive Si–OH groups and the overcoming of repulsive interactions between silicate species are prerequisites for the aggregation of colloidal silicate particles and gel formation and are mainly governed by the pH.

(1) $$\equiv \mathrm{Si}-O^{-}\cdots {\;\mathrm{Na}}_{\left(\mathrm{aq}\right)}^{+}+{\;\mathrm{HX}}_{\left(\mathrm{aq}\right)}\;\to \;\equiv \mathrm{Si}-{\mathrm{OH}}_{\left(\mathrm{aq}\right)}+{\;\mathrm{NaX}}_{\left(\mathrm{aq}\right)}$$

In the initial composite design, the waterglass binder was exposed to CO_2_ in ambient air for an extended period (Figure 1b), allowing CO_2_ to partially dissolve into the waterglass to form carbonic acid (Equation (2)). Carbonic acid introduces hydrogen ions into the solution through dissociation (Equations (3) and (4)), which then react with silicate species, resulting in the formation of a silica gel. Silica gel binds adjacent bioactive glass particles together, leading to the hardening and the setting of the composite. Notably, over this lengthy period (>10 days), all the water in the waterglass evaporates, leaving any formed gel and any remaining binder fully dried. The reaction between waterglass and CO_2_ is inherently slow because carbon dioxide is present in the atmosphere in very small amounts (~0.04%).

(2) $${\mathrm{CO}}_{2\;\left(g\right)}+{\;H}_{2}O_{\left(l\right)}\;↔{\;H}_{2}{\mathrm{CO}}_{3\;\left(\mathrm{aq}\right)}$$

(3) $$H_{2}{\mathrm{CO}}_{3\;\left(\mathrm{aq}\right)}\;↔{\;\mathrm{HCO}}_{3\;\left(\mathrm{aq}\right)}^{-}+{\;H}^{+}$$

(4) $${\mathrm{HCO}}_{3\;\left(\mathrm{aq}\right)}^{-}\;↔{\;\mathrm{CO}}_{3\;\left(\mathrm{aq}\right)}^{2-}+{\;H}^{+}$$

Preliminary experiments (see Appendix A) showed that applying concentrated CO_2_ gas could reduce the composite’s setting time to under ten minutes while maintaining shape retention and stability. However, this method had significant drawbacks:

- Lack of repeatability;
- Mass transport limitations at the surface;
- Restricted to a narrow pH range, despite silica gel properties being pH-dependent.

Therefore, the project shifted to using acid solutions as initiators mixed with waterglass, replacing CO_2_ gas, to accelerate the setting reaction (Figure 1c). This volume-independent approach ensures even dispersion of hydrogen ions, promoting uniform gelation throughout the scaffold. Consequently, the setting times of bioactive glass–waterglass composite scaffolds are primarily governed by the gelation time of the waterglass, which is the focus of this study.

The gelation time of sodium silicate solutions can be controlled and accelerated by controlling the parameters influencing silicate polymerization and aggregation, and consequently gelation kinetics in these solutions. These parameters include pH, waterglass concentration or dilution rate, the SiO_2_/Na_2_O molar ratio of waterglass, the presence of salts, type of acid initiator, and temperature, which is comprehensively discussed in our recent review paper [16]. Considering the degree of effectiveness and the conditions specific to our target application (bone tissue scaffolds), we narrowed down the parameters investigated here to these main three:

1. pH: pH is a key parameter controlling gelation in silica–water systems. Although many studies have examined the effect of pH on waterglass gelation time, they are typically limited to narrow pH ranges of 1–2 units [17,18,19]. A comprehensive study conducted by Iler [20] in the 1970s focused on silica–water systems in general, but not specifically on waterglass, leaving the general trend for gelation time across the entire pH range for waterglass uncertain. Detailed data on the relationship between gelation time and pH are essential for precisely modeling and controlling the gelation process, which is critical for tailoring the properties of the resulting gel. Although applying soluble silicates to biomaterials limits us to a pH range safe for human tissue, data on gelation time across the whole pH range are the missing puzzle piece for developing gels from waterglass. This information is crucial for optimizing the gelation process, regardless of the target application.
2. Type of acid initiator: various acids—both strong (such as hydrochloric acid [21], nitric acid [22], phosphoric acid [23], and formic acid [24]) and weak (such as boric acid [25], acetic acid [26] and carbonic acid [27])—are used for silica gel formation from waterglass for different applications. However, the rationale behind the selection of a given acid as an initiator is rarely explained in the literature and appears to have been chosen arbitrarily. Studies using multiple acid initiators for silica gels [28,29] typically fail to maintain constant pH, making it unclear whether the type of acid initiator affects gelation time beyond its influence on pH. Here, we seek to elucidate the effects of acid initiators by controlling and fixing other independent variables.
3. Waterglass concentration: Gelation is known to accelerate with an increase in sodium silicate content in water [16]. While higher concentrations initially promise shorter gelation times, adjusting waterglass concentrations offers flexibility to achieve specific gelation times across varying pH conditions. Therefore, we investigate the impact of initial waterglass concentration on gelation kinetics to optimize the gelation processes tailored to bone tissue composite scaffolds.

With the goal of eliminating the “trial and error” approach in manufacturing silica gel from waterglass and improving structural control in silica gels derived from these solutions, our discussion focuses on the following points:

- How should pH and waterglass concentration be adjusted to achieve a target gelation time that is practical for making composite bone scaffolds?
- Can gels with the same gelation time be produced under different processing conditions (i.e., pH, waterglass concentration, type of acid initiator)? How do these conditions affect the final properties of the gels?
- At a fixed gelation time, does the type of acid initiator influence the final properties of the gel?
- Can the appearance and optical properties of the gels provide insights into the sol–gel kinetics and the structure of the final gels?

This manuscript is the first in a series of studies focused on elucidating the processing–structure–property relationships in waterglass-based silica gels. Subsequent publications will explore molecular and microstructural characteristics, as well as mechanical properties of silica gels for optimizing the processing conditions and controlling the final properties for bone tissue engineering applications.

## 2. Results and Discussion

### 2.1. Gelation Time

**Figure 2:**
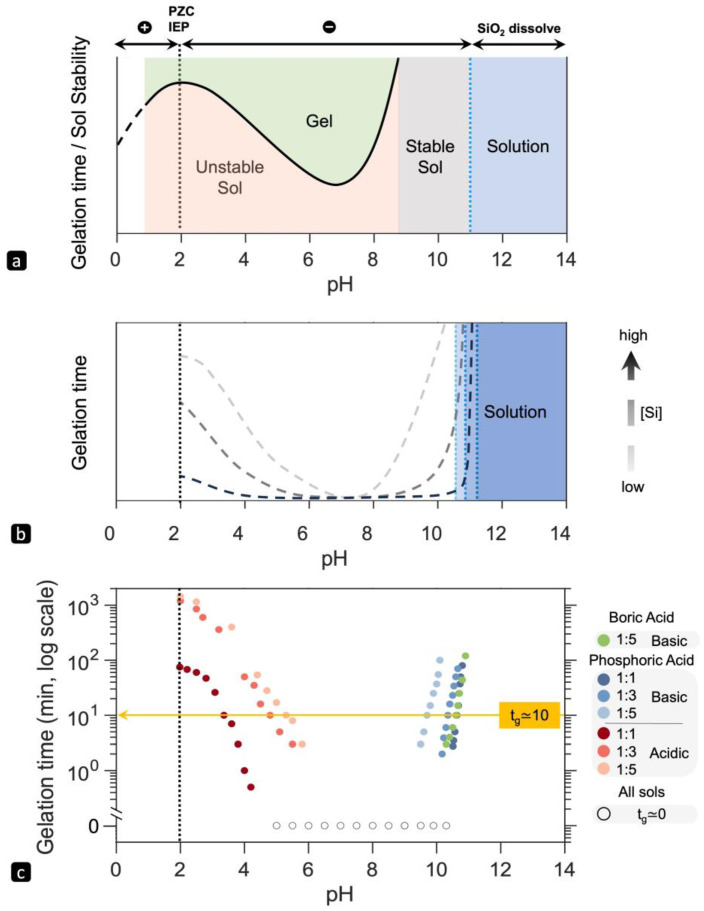
(**a**) the general trend for gelation time/sol stability versus pH for silica water systems, redrawn from [20]; (**b**) the general trend for gelation time versus pH for waterglass solution of different concentrations observed in this study; (**c**) semi-log graph of gelation time versus pH for specimens made of boric acid and phosphoric acid and different initial waterglass concentrations. The target gelation time of 10 min. is marked by the yellow arrow, which intersects the various solutions at different pH values, and hence permits multiple processing routes to fabricate a gel that forms in 10 min. The vertical dashed line presents the point of zero charge (PZC) and isoelectric point (IEP).

pH is the most critical parameter influencing sol–gel reactions in silicate solutions. Figure 2a schematically illustrates the general stability/gelation time trend proposed by Iler [20] for silica–water systems across the whole pH range. This complex behavior indicates that electrostatic interactions alone cannot primarily dictate stability and particle aggregation/bonding in silicate solutions; instead, the silanol functionality and condensation reaction rate (initiated by OH^−^ or H^+^), play a more significant role in the gelation of silicate solutions.

The pH-dependence of gelation time for sodium silicate solutions of different concentrations, acid-initiated with phosphoric acid and boric acid was measured using the tube inversion method. Our findings broadly agree with the trend observed in silica–water systems (Figure 2b). However, we did not observe the “stable sol” region. It is important to clarify that the terms “stable sol” (referring to colloidal dispersion) and “solution” (referring to molecular/ionic dispersion) are conventionally used for monomeric silicate species in silicate solutions. Waterglass, however, contains both ionic species and colloids depending on the SiO_2_/Na_2_O ratio and is not a true solution despite common usage of the term. The disappearance of the stable sol region in waterglass implies that colloidal particles cannot grow below pH ≈ 11 unless they aggregate at some point. Additionally, waterglass exhibits a rapid or immediate gelation zone instead of a minimum gelation point.

In sodium silicate solutions, the presence of sodium cations facilitates closer proximity among silicate particles, likely due to the charge screening effect of the cations and implications from the electric double layer theory (see reference [30] for further details).

The closer proximity of colloidal silicate particles in waterglass widens the minimum gelation zone. As shown in Figure 2b, both the width of the “rapid gelation” zone and the slope of the graph (indicating the dependency of gel time on pH) appear to be influenced by silica concentration. Specifically, as the dilution rate increases, transitioning from a WG:Water weight ratio of 1:1 to 1:5, (i) the slope in the basic region decreases, (ii) the “rapid gelation” zone narrows, and (iii) the maximum gelation time in the acidic region increases. Increasing silica concentrations (equivalent to decreasing WG:Water weight ratio) enhances gelation by fostering the formation of more silicate particles, and consequently increasing the likelihood of collisions. These collisions can result in the formation of siloxane bridges through chemical interactions or lead to physical interactions such as hydrogen bonding. Therefore, higher silica concentrations increase the probability of particles aggregating through chemical or physical mechanisms, which results in broadening the “rapid gelation” zone and a decrease in max gelation time at pH ≈ 2. These findings are in agreement with previous studies investigating the effect of pH on the gelation time of sodium silicate solutions [17,19,31,32], although none of these studies explored such a wide pH range.

The relationship between gel time and pH for all the gel specimens is shown on a semi-logarithmic scale in Figure 2c. Using phosphoric acid, a strong acid, gels can be prepared across the entire pH spectrum of 2-11. However, with boric acid, a weaker acid, the pH can only be lowered into the basic range. In the basic region, the gel time versus pH changes exponentially, moving towards near-infinite gelation time or stable solution with increasing pH. In contrast, in the acidic region, the curves fit to fourth-order polynomials, reaching a maximum as the pH approaches 2. The fitted equations in Table 1 reveal that the constant parameters in fitted equations vary with waterglass concentration, indicating that gelation kinetics change with concentration. By determining the relationship between the coefficients and waterglass concentration from experimental data, we can predict the gelation time for any given concentration and pH.

**Figure 3:**
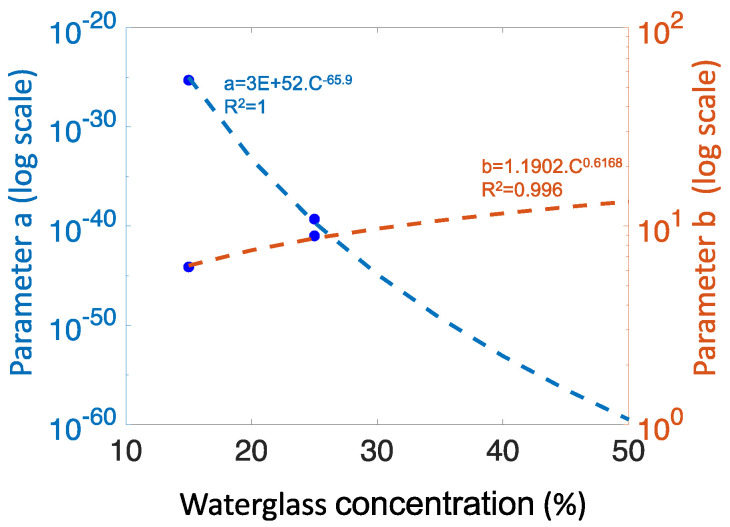
Dependency of parameters a and b on waterglass concentration. These parameters are part of the fitted equation describing the relationship between gelation time and pH for basic gels (Table 1). The fitted equations and R-values are shown on the graph.

In the basic region, the parameters a and b in the general equation were found to be functions of waterglass concentration (*C*, wt%) (Figure 3). The general formula of gelation time (*t_g_*) versus pH in the basic region, and the relationship between the equation parameters a and b with concentration can be expressed as follows:

(5) $$t_{g}=a\cdot e^{b\cdot \mathrm{pH}}$$

where

(6) $$\mathrm{loga}=\left(4E-80\right)\cdot C^{-65.9}$$

(7) $$\mathrm{logb}=\left(20.384\right)\cdot C^{0.6169}$$

When comparing boric acid and phosphoric acid in the basic region, we observe that the a and b parameters for BA fitted equations are very close to that of PA 1:5. This similarity could be linked to the fact that, although the initial WG:Water ratio of the silicate solution was 1:1 for this sample, it increased to 1:5 by the large amount of water introduced through addition of BA. It appears that there is a general exponential equation for gelation time versus pH in the basic region, and its parameters depend solely on waterglass concentration (and not on the type of acid initiator used). It should be noted that the SiO_2_/Na_2_O ratio of waterglass is constant in these observations, so the general trend is limited to this condition. If the SiO_2_/Na_2_O ratio were to vary, the a and b parameters may also change. Overall, the impact of pH and WG concentration on gelation time is significantly more influential than the type of acid used.

Similarly, in the acidic region, the constant parameters of the general equation for gelation time versus pH vary with waterglass concentration. Due to the high number of parameters and the complexity of the equations, we could not fit the relation with high certainty. Fitting this relationship more accurately would require more data points and the aid of computational methods, which could be the focus of another project. However, the maximum gelation time at pH = 2 was also a function of waterglass concentration (*C*, wt%) and followed a second-order polynomial relationship. This relation can be expressed for maximum gelation time (t_g_^max^) as follows:

(8) $$t_{g}^{\max}=-6000C^{2}+1575$$

The second-order polynomial relationships with concentration suggest that the influence of concentration on gelation kinetics is complex and potentially includes interactions between different contributing factors.

**Figure 4:**
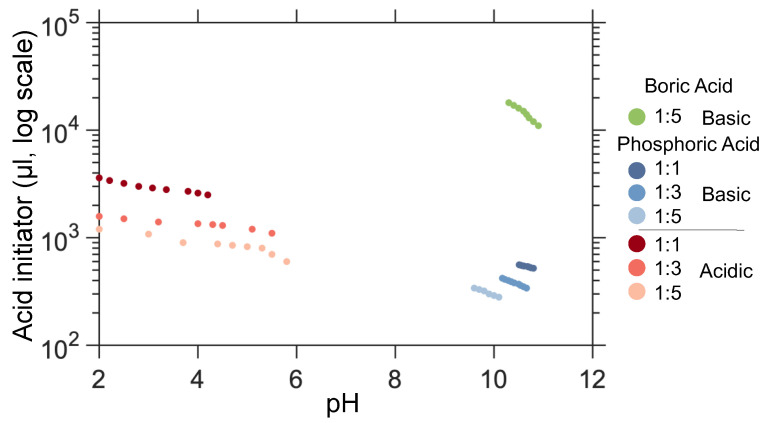
The graph shows the amount of acid initiators (boric acid and phosphoric acid) needed to adjust the pH of silica gels made from waterglass at different concentrations. For clarity, the y-axis is presented in a logarithmic scale.

The amount of acid required to adjust the pH with BA and PA is illustrated in Figure 4. As expected, since BA is much weaker than PA, the required amount of BA is 28–38 times greater (depending on WG concentration) than the amount of PA needed to reach the same pH. Additionally, as the WG content in the initial solution decreases, the amount of acid needed to lower the pH also decreases. The pH is very sensitive to slight changes in the amount of acid in the PA 1:1 sample, and this sensitivity decreases as the WG concentration decreases or the pH moves into the acidic region. Consequently, controlling the pH and gelation time was most challenging with the PA 1:1 sample. Although the BA sample had the lowest pH sensitivity to changes in acid content, it cannot be considered the easiest to control. The BA pH sensitivity is because the WG-to-water ratio changes abruptly with the addition of acid, leading to fluctuations and making predictions more difficult.

Generally, the results indicate that achieving a desired gelation time is feasible across both acidic and basic pH ranges. Referring to Figure 2c, we observe that the target gelation time of 10 min is met by one point using BA in the basic region, and two points using PA at each of three different concentrations in both acidic and basic regions, totaling seven specimens, as detailed in Table 2. Moving forward, we will proceed with these seven samples, all of which exhibit a gelation time of 10 min, making them suitable for the desired clinical applications.

### 2.2. Optical Properties

During the sol–gel process, the colloidal particles present in the sol gradually form a network that evolves into a gel structure, which traps the surrounding solvent. These structural changes are also reflected in the optical properties of the resulting gels, leading to variations from transparent to translucent and even opaque appearances. Therefore, investigating the optical characteristics throughout the sol–gel transition can provide invaluable insights into the structural evolution during gelation. This investigation was performed through both visual observations and quantitative techniques such as UV/VIS spectrophotometry.

#### 2.2.1. Visual Changes

**Figure 5:**
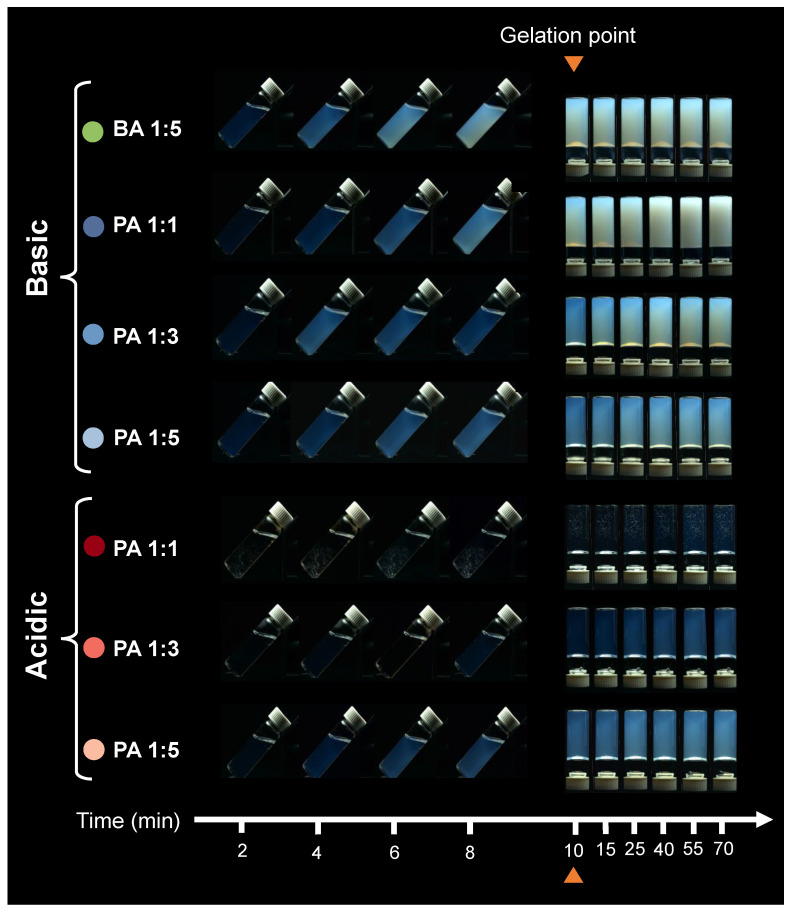
Macrograph of silica gels during sol–gel transition and post-gelation over time. All images were taken at constant white balance and exposure conditions and the blue cast to the images is true to what was usually observed. Gels have varying degrees of transparency, ranging from a clear appearance to a blue cast and opaque.

Figure 5 demonstrates the sol–gel progression in acidic and basic gels prepared using PA and BA, spanning up to 60 min after gelation. Gelation of all samples occurred at the 10 min mark, clearly indicated by the inversion method. Initially, the sols appeared clear and colorless after introducing the acid. However, during and after transforming to gel, the gels exhibited distinct optical behaviors influenced by pH and silica concentration. Basic gels transitioned from transparent to translucent, with a decrease in transparency persisting post-gelation. The PA 1:1 basic specimen, in particular, became opaque. A similar trend was observed in BA basic gel, highlighting that the effect of pH on the gelation process was more significant than the type of acid initiator used. Acidic gels were notably more translucent in contrast to basic gels. For instance, the PA 1:1 acidic sample remained completely transparent up to the gelation point, although it contained some entrained gas bubbles. The reaction between WG and the acid initiator is exothermic in the acidic region, generating heat that can be felt when handling the vial, and these bubbles are likely a result of this exothermic reaction inducing vapourization of the solvent.

Overall, a decrease in light transmission was observed across all samples, which continued after gelation, with all samples exhibiting a faint blue color along with reduced transparency. The differences in the appearance of basic gels in contrast to acidic gels suggest microstructural differences between these two groups of gels.

#### 2.2.2. Tyndall Effect

Waterglass consists of a wide range, and physical size, of species ranging from monomers to oligomers and all the way up to colloids. Investigations by dynamic light scattering and small-angle X-ray scattering have reported a wide range of colloidal particle sizes in waterglass—0.6 nm to 600 nm—and uncertainties about their size range continue to exist [33,34]. Upon acidifying waterglass, the silicate species, and colloidal particles, continue to grow until they are no longer stable and undergo aggregation through physical or chemical interactions into fractal structures [35,36]—thus resulting in the gelation of the system. The final size of the aggregates formed by these particles directly affects the microstructure and porosity of the final gel; the physical, chemical, and mechanical properties depend on the microstructure. The size of colloidal silicate particles and aggregates also affects how they interact with, and scatter, light. By observing the Tyndall effect, one can indirectly study the size of these particles and aggregates, thereby gaining insights into the differences in the final structure of gels formed under different processing conditions.

According to Rayleigh, the relative intensity of the light scattered at right angles to the axis of illumination by dilute sols of constant volume concentration, can be expressed as [37]:

(9) $$I=\frac{\mathrm{kcv}}{{\rho \;\lambda }^{4}}$$

where k represents the Boltzmann constant; c is the concentration of the colloidal solution; v represents the volume of the scattering particles; ρ is the density of the particles; and λ denotes the wavelength of the incident light.

Equation (9) indicates that, at a fixed wavelength, the intensity of the scattered light is directly proportional to the average volume of the particles. However, this relationship assumes certain conditions, such as particles being optically isotropic and randomly distributed. In concentrated colloidal solutions like ours, not all these assumptions hold true, complicating the straightforward relationship between intensity and particle size, and a definitive solution to this issue remains unresolved [38]. Overall, the nature of particles—specifically their size, shape, refractive index, and surface characteristics—all play significant roles in scattering behavior. Nonetheless, it is widely accepted that an increase in either the number, or size, of particles typically results in higher intensity of the scattered light. Therefore, we anticipate that the size and arrangement of silicate particles/aggregates in our system directly influence the amount of light scattered by them. However, finding a mathematical model for quantitatively relating these two parameters would be a challenge and was not in the scope of this work.

**Figure 6:**
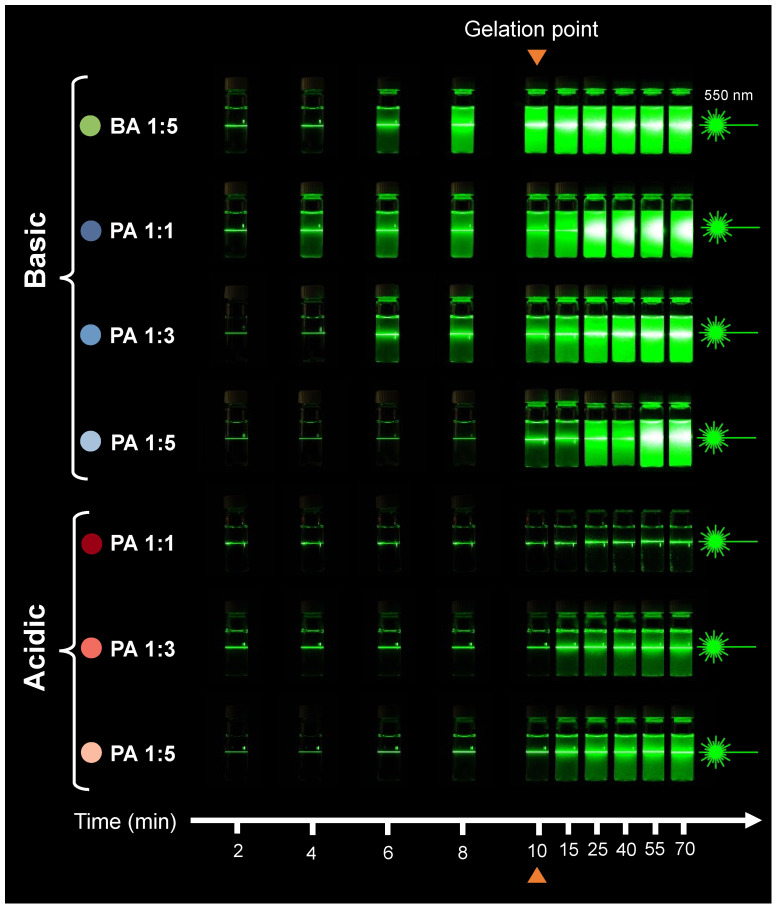
Macrographs showing the Tyndall effect of silica gels during sol–gel transition and post-gelation over time while shining a 550 nm green through the specimens. The laser is causing a reflection above the laser line from the top surface of sol–gel meniscus, or oven the top cap on the vials in some cases. All images were taken at constant white balance and exposure conditions and the green reflection is true to what was usually observed. The changes in light scattering, indicated by the intensity and amount of reflected green light, are inversely correlated with light transmittance in Figure 5. This observation suggests that the light not transmitted through the sample is scattering rather than being absorbed.

A green 550 nm laser was used to qualitatively investigate light scattering and the Tyndall effect during the sol–gel transition and post-gelation across all seven specimen types (Figure 6). Our observations yielded the following insights:

- Initially, all samples exhibited the Tyndall effect to some degree, confirming the colloidal nature of the initial waterglass solution.
- The overall trend of the light scattering in our samples closely mirrors that seen in Figure 5, indicating that the reduction in light transmission results from increased scattering by colloidal particles or aggregates.
- Over time, after introducing the acid-initiator, the intensity of scattered light in each sample increases. This increased scattering is evident from both the increased brightness of the beam path and the glow that expands around it, often illuminating the entire vial. This glow is attributed to multiple scattering centers within the specimen, which redistributes light in various directions, making scattered light visible beyond the direct laser beam path. This scattering phenomenon suggests the formation of new colloidal particles, an increase in colloidal particles, or both.

Specifically, in the case of acidic PA 1:1 gel, the slight increase in the density of the beam path with no glow suggests that particles or aggregates were small, and light was not re-scattered from the surroundings.

#### 2.2.3. UV/VIS Spectrophotometry

**Figure 7:**
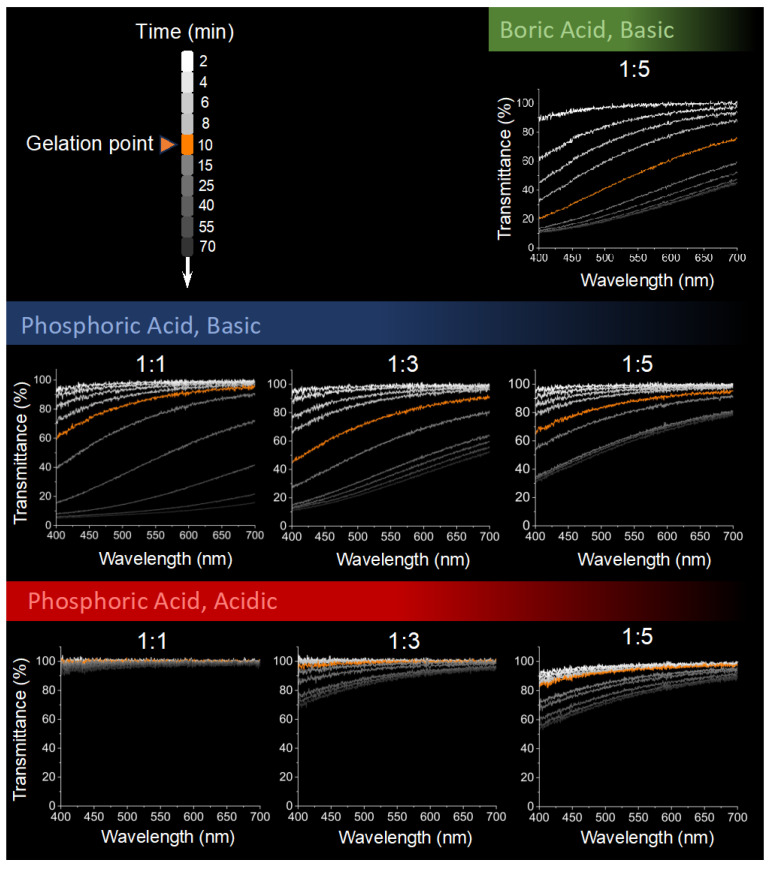
UV/VIS spectra of silica gels during sol–gel transition. The spectra show light transmittance in the visible light region (400–700 nm) at various time intervals after mixing waterglass solutions of different concentrations with boric acid and phosphoric acid. The measurements were taken at the gelation point and up to 60 min after gelation. The figures show decreased light transmittance over time for all gels and less light scattering for acidic gels compared to basic gels.

The UV/VIS spectra of all the gels during the sol–gel transition, at the gel point, and up to 60 min after gelation are illustrated in Figure 7 within the visible region. Several observations are evident at first glance:

- There is an increase in transmittance with increasing wavelength, contributing to the bluish tint observed.
- The overall transmittance decreases with time within each sample, resembling the photos in Figure 5.
- There is a distinct difference between acidic and basic gels in their ability to transmit light, with acidic gels having a higher %T, regardless of the type of acid initiator used. However, the difference in the light transmittance behavior diminishes as the dilution rate increases.
- Basic gels show an increase in transmittance with decreasing silica concentration, whereas acidic gels exhibit a decrease in transmittance with dilution.

The BA basic gels’ transmittance spectra over time are similar to those of the PA 1:3 gel. Generally, more light is transmitted as the wavelength increases from 400 to 700 nm, with the difference being more pronounced in basic gels compared to acidic gels. According to Equation (9), the intensity of the scattered light is inversely proportional to the fourth power of the wavelength. If we consider the extinction to be solely due to light scattering and accept that the intensity of the incident light equals the sum of the transmitted and scattered light, then %T should be directly proportional to the fourth power of the wavelength. From Figure 7, there is a general agreement with this direct proportion; however, the light transmittance dependency on wavelength is not constant and is influenced by concentration, pH, and time.

However, for example, at the gelation point, the ratio of %T at 700 nm to 400 nm varies between approximately 1.1 and 5, ranging from almost independent of frequency to dependency on the third power (~5.35) of the wavelength. Thus, as mentioned in the previous section, the relationship between %T and wavelength is more complex than is suggested by Equation (9), due to the complex nature of our system, which includes different ions, oligomeric and polymeric species, particles, aggregates, and pores. Nonetheless, the light at 400 nm is more scattered than at 700 nm, explaining the bluish tint observed in the specimens (Figure 5).

Combining the results from Figure 5, Figure 6 and Figure 7, the general decline in transmission over time is attributed to the increased light scattering intensity due to the formation and/or growth of colloidal silicate particles and aggregates, as well as light scattered from gel–pore interfaces. The continued change in %T post-gelation indicates that the system remains dynamic, with ongoing particle growth and changes in pore size, a process known as syneresis [16]. Moreover, the differences in optical properties between acidic and basic gels suggest pH-dependent cluster–cluster aggregation. For example, in the PA 1:1 acidic gel, the sample shows more than 90% transmittance over the 400–700 nm wavelength range at all times, with a glass-like transparent appearance. In contrast, the PA 1:1 basic gel shows a decrease in %T to less than 10% over the same wavelength range, with an opaque appearance 60 min after gelation. This difference in behavior indicates varying fractal and network structures in these gels, likely due to differences in the size and connectivity of the silicate particles forming them.

Finally, the light transmittance in the basic gels is primarily influenced by pH and WG concentration, rather than the type of acid initiator used. The transmission spectra of the BA basic gel over time resemble those of the PA 1:3 gel. Despite starting with a solution concentration of 1:1, which increased to 1:5 after adding the acid initiator solution, it exhibits transmission characteristics that align more closely with the average of these concentrations. The gelation time–pH dependency, however, resembles that of the PA 1:1 sample, indicating variability in predictability and control.

### 2.3. What Do Optical Properties Reveal About Gelation Kinetics?

Chemical kinetics concerned with reaction rates are typically defined as the change in concentration over reactant or product per unit time. Gelation, however, is not a simple chemical reaction in which two, or multiple reactants, are consumed to form product(s). During the sol–gel transition, silicate particles form and/or grow through polymerization–depolymerization reactions and these suspended particles aggregate and extend throughout the system, forming a gel network. Aggregation kinetics are controlled by particle–particle attachment efficiency, which is the fraction of particle–particle collisions resulting in attachment (through either physical or chemical interactions) and is dependent on solution chemistry, and the particle–particle collision frequency [39]. If we reasonably assume and accept that the transmitted light decay/increased light scattering is caused by the change in size, shape, and arrangement of colloidal silicate particles, then we can conclude that monitoring the change in transmittance over time allows us to study the gelation kinetics.

**Figure 8:**
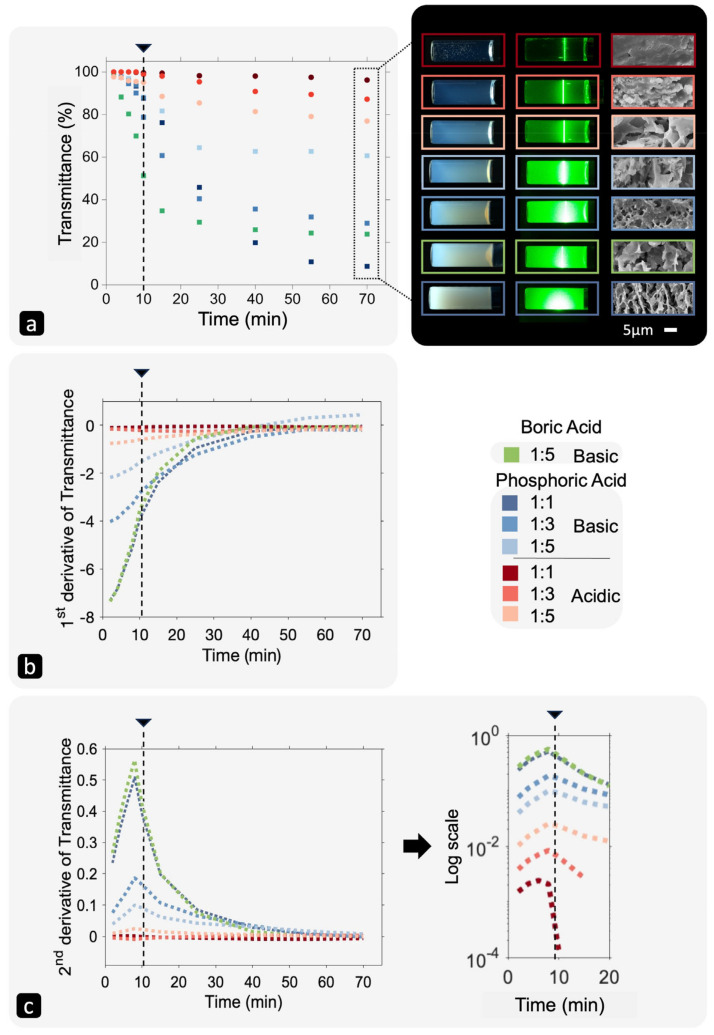
(**a**) Light transmittance at an average wavelength of 550 nm over time for silica gels made with phosphoric acid and boric acid at different waterglass concentrations (**left**), light transmittance macrographs, Tyndall effect macrographs obtained by shining a 550 nm laser, and SEM micrographs of the gels, taken 60 min post-gelation (**right**). Although all gels share the same gelation time of 10 min, they exhibit distinctly different optical properties and microstructures based on processing conditions. (**b**) The first derivative of light transmittance of silica gels versus time, showing an inflection point around the gelation point. (**c**) The second derivative of light transmittance of silica gels versus time, used to more clearly identify changes in the light transmittance rate. The maxima at ~8 min indicate the onset of gelation. The dashed line and arrow mark the gelation point at 10 min by tube inversion method in all figures.

The light transmittance at an average wavelength of 550 nm is plotted against time for all seven samples in Figure 8a. The decrease in light transmission continues after the gelation point due to syneresis, or aging of the gels. When the light transmittance stops changing, it usually indicates that the reaction is complete. As shown in Figure 8a, the curves do not reach a plateau during the testing duration, indicating that the structural development responsible for the light scattering (syneresis process) is ongoing. However, their shape suggests that they tend to reach a plateau over longer periods.

The %T curves were best fit to a third-order polynomial equation (Table 3). The non-linear model captured varying rates of change in light transmittance, indicating different stages of particle formation, growth, aggregation, and gel network development. Two stages in Figure 8a were observed over time.

Firstly, rapid changes in light transmission up to the gelation point, followed by slower changes afterward. This change in slope near the gelation point is evident in Figure 8b, where the first derivative of the fitted equations is plotted. During the sol–gel transition, the system undergoes massive changes. The colloidal particles move, form connections, and create a 3D network of aggregates and water-filled pores. Secondly, after gelation, the gel network continues to grow and densify, but the rate of change is much slower compared to before the gelation point. The mobility of particles within the gel decreases significantly, leading to a slower change in light transmission properties.

The fact that the light transmission of gels over time can be fit by a polynomial equation raises the question of such an equation’s suitability for gelation kinetics, and if it is chemically, or physically, meaningful for the multitude of processes occurring during the sol–gel transition. Several kinetic models were proposed to describe the sol–gel process, such as Kinetic Monte Carlo Simulations [40], as well as diffusion-limited cluster aggregation (DLCA) and reaction-limited cluster aggregation (RLCA) [35,41]. Overall, considering the heterogeneous nature of gelation, the involvement of multiple stages and mechanisms, various influencing factors, the dynamic and non-equilibrium characteristics, as well as the distribution of particle sizes and shapes, it is not surprising that no exact analytical solution can be found for treating all kinetic gelation processes.

The reaction or gelation rate is usually described using power-law or exponential equations and polynomial equations for kinetics are less common. However, kinetic polynomials were proposed as the most generalized form of kinetic equation for complex reactions and is proved to be consistent from a thermodynamic point of view [42,43]. Unfortunately, as Marin et al. [44] pointed out, the reasoning and details provided in the kinetic polynomial literature are mostly in favor of mathematical optimization and not very comprehensive of the chemical phenomena being described. Still, there are a few studies in which gelation time was reported to be a polynomial function of the polymer and cross-linker/polymer ratio [45,46]. While a polynomial fit is not the most traditional model for gelation kinetics, its flexibility in fitting data with multiple inflection points seems appropriate for complex systems and suggests additional factors are at play. It is important to note that studying gelation kinetics is usually performed by measuring various properties during the sol–gel transition such as particle size [47,48] or viscosity [31] or like here, light transmission over time. However, these properties may exhibit different relationships with the gelation process itself. For instance, while viscosity in an acidic gel may sharply increase during gelation, light transmission might show minimal change. Therefore, expecting a single form of equation to universally describe *all* aspects of gelation would be impractical. While there exists a known link between fractal dimension and the type of cluster–cluster aggregation (DLCA vs. RLCA) [49], no universal mathematical relationship exists for aggregation and light transmission.

Another reason to scrutinize a polynomial model of light transmittance over time is the model’s potential to predict the gelation point. There are a number of studies that have used differences in light transmission/scattering to define the gelation point [50,51,52]. For instance, Boschel and Roggendorf [50] found that the first derivative of transmission obtained by UV/VIS spectroscopy may be used as a useful indicator of the gel point in borosilicate gels. Derivative spectrophotometry involves differentiating a normal spectrum and was shown to be an effective method for enhancing the time resolution [53]. This technique highlights subtle spectral features by presenting them in a visually more accessible way and reducing the effect of spectral background interference. 

Figure 8c reveals that interestingly, the second derivative of transmittance versus time of gels shows a maximum at around 8 min, which closely aligns with the 10 min gelation time obtained by the tube inversion method. This maximum in the second derivate curve suggests a rapid change, or inflection, in the rate of gelation at the gelation point, which is also evident in the first derivate, though less obvious. The slight discrepancy in timing between the two methods of gel-point determination arises because the tube inversion method identifies the point at which the gel becomes completely rigid, while the point obtained through light transmission may signify the onset of gelation.

The PA 1:1 acidic gel stands out as the only sample that remains transparent even after gelation. However, even for this sample, there is a subtle change in the slope at the gelation point. This change can be intensified in a semi-log graph, as illustrated at right in Figure 8c. Continuing the derivate to higher orders is another way to improve time resolution. It should be noted that if using the fourth derivative of transmittance as a function of time, the gelation point will occur when the fourth derivative equals zero—corresponding to the maximum or minimum of the third derivative. Overall, the results suggest that light transmission over time during the sol–gel transition can be used to detect the gelation point, marked by a significant decrease in rate of change of light transmission. However, depending on the processing condition, this change may be easily observed in the raw data set, or multiple derivates may be needed to improve the time resolution and, therefore, precise determination of the gelation point.

### 2.4. Relationships between Gel Structure and Optical Properties

How do the size of silicate particles, aggregates, pores, and the overall gel network arrangement affect the optical properties? In this section, we aim to establish connections between qualitative and quantitative observations of optical properties and the microstructure of the gels.

#### 2.4.1. Acidic vs. Basic Gels

As mentioned earlier, acidic gels generally scatter less light compared to basic gels, which is consistent with prior studies [54]. At the highest silica concentration (1:1 ratio), basic and acidic gels represent two extremes: 60 min after gelation, the PA 1:1 basic gel becomes practically opaque (8.7% T at 550 nm), while the PA 1:1 acidic gel remains largely transparent (96.2% T at 550 nm). These gels also exhibit the lowest and highest pH among all samples (3.36 for acidic and 10.63 for basic). The difference in transparency at similar wavelengths is attributed to how the silicate aggregates form a 3D network. SEM micrographs clearly show the difference in these gels’ microstructures (see Figure 8): the PA 1:1 acidic gel is dense and compact, while the PA 1:1 basic gel has a uniform porous structure with pore diameters ~5 µm. It is important to note that the PA 1:1 acidic specimen cannot be free of pores; both 1:1 hydrogels contain the same amount of water trapped inside the pores before freeze-drying. Therefore, the pores in the acidic gel are too small to be seen clearly. Moreover, the pores are not just filled with pure water; they may contain silicate monomers and/or oligomers, sodium ions, and ions from the acid, which remain inside the pores after water evaporation, filling some of the space. The compact structure of the PA 1:1 acidic gel gives it an almost glass-like appearance, suggesting that the refractive index of its aggregates closely matches that of the solvent in the pores. The compactness of a 3D structure made of almost spherical particles forming aggregates depends on many factors, including particle and aggregate size, particle and aggregate size distribution, and the type and amount of bonding between the particles. Thus, the contribution of all of these parameters made acidic gels glass-like features, likely composed of smaller particles that form smaller aggregates and smaller pores, resulting in less light scattering. In contrast, the PA 1:1 basic gel is made of larger particles and larger aggregates with larger pores in between that scatter light more effectively, resulting in reduced transmission and increased opacity. Larger aggregates also contribute to the formation of larger pores compared to acidic gels. The difference in the initial size of silicate particles in these gels is influenced by pH, which governs the polymerization–depolymerization reactions in waterglass, thus influencing the gel’s microstructure and optical properties.

#### 2.4.2. Effect of Concentration and Type of Acid Initiator

Two opposing trends are observed for acidic and basic gels: (1) as the water content increases (waterglass:water ratio decreases), light transmission increases in basic gels but decreases in acidic gels. In other words, basic gels scatter less light while acidic gels scatter more light as silica concentration decreases. It appears that at some point above, with a waterglass:water ratio of 1:5, the light scattering of basic and acidic gels tends to converge. This opposing trend is evident in Figure 8a, where the %T, appearance, Tyndall effect and SEM micrograph of gels can be compared 60 min after gelation. When examining the gels’ microstructures, we see that gels in both acidic and basic groups become more porous and less uniform upon dilution. So why do these trends appear to be opposite?

Concentration is not the only factor changing herein; in each group, pH is also adjusted with concentration to achieve the same gelation time across all specimens (recall Figure 3). However, the trend of pH change in the two groups is opposite; in the basic group, pH decreases upon dilution, while in the acidic group, pH increases upon dilution. This difference in pH adjustment affects the particle size, contributing to the observed optical-related trends.

Particles and aggregates are not the only scatterers in these sol–gel systems. Once past the gelation point, pores form and also scatter light. The level of porosity (pore volume fraction) and the pore size, and size distribution, as well as the presence of other phases with different optical properties in the pores, all affect light scattering. Although both pore size and porosity increase from a waterglass ratio of 1:1 to 1:5 in both acidic and basic gels, the rate of increase is dissimilar due to different starting pore sizes. For instance, the pores in the acidic 1:3 gel are still much smaller than those in the basic 1:3 gel. However, both the 1:5 acidic and basic gels exhibit similar microstructures and pore sizes (>10 µm).

According to Hříbalová and Pabst [38], the van de Hulst approximation [55] is the only model that predicts both the decrease in light transmittance with increasing pore size in the small-size region (i.e., smaller than the wavelength) and the increase in transmittance with increasing pore size in the large-size region (i.e., larger than the wavelength). This model and experimental data show that light transmittance is minimal when the pore size is around the wavelength of the light used, with a steep increase towards maximum transmittance in the small-size region. For larger pore sizes, the increase in transmittance is more gradual and does not reach maximum levels.

In basic gels, the pores are at least 10 times larger than the wavelength, so according to the van de Hulst approximation, light scattering by pores decreases with increasing pore size from a waterglass ratio of 1:1 to 1:5. This trend in pore size explains the increase in total light transmission in basic samples with dilution, as both scattering by particles—due to the decrease in silica concentration and the number of particles—and scattering by pores decrease, regardless of any changes in particle size caused by the pH decrease (~1). In acidic gels, the situation is different. The pores in acidic 1:1 and 1:3 gels fall into the small-size region where the pore size-scattering slope is negative, causing the 1:3 gel, with larger pores, to scatter more light compared to the 1:1 gel. In the 1:5 acidic gel, the pores are in the large-size region and scatter light similarly to the basic 1:5 gel, more than both the 1:1 and the 1:3 gels. The acidic 1:5 gel scatters more light even before gelation, which could not be related to pores. This effect is likely due to the large difference in particle size in the 1:5 gel compared to the other two because of the significant pH difference (~2).

## 3. Conclusions

In this study, we demonstrated that the gelation kinetics of sodium silicate solutions could be controlled by adjusting the processing conditions. To optimize waterglass gelation kinetics and the final gel microstructure for enhanced performance as a binder phase in composite bone scaffolds, the effects of pH, waterglass concentration, and type of acid initiator were investigated. The key findings are as follows:

- Gelation time changed exponentially with pH in the basic region and fit third-order polynomials in the acidic region, peaking near pH 2. The fitted equation parameters varied with waterglass concentration, allowing prediction of gelation time for any given concentration and pH.
- A wide range of target gelation times could be achieved in both acidic and basic regions. The specific pH for the target gelation time could be adjusted toward neutral pH by decreasing the initial waterglass concentration.
- Studying the optical properties of gels, while not directly critical in bone tissue engineering, provided insights into gelation kinetics and microstructure, which influence essential properties such as setting time, porosity, and presumably mechanical strength.
- With BA, being weaker than PA, 28–38 times more volume of acid was needed to adjust the pH and only basic gels could be made. Overall, the gelation kinetics, optical properties and microstructure of BA basic gel were similar to what was observed for PA basic gels, highlighting that the effect of pH and waterglass concentration on the gelation process is more significant than the type of acid initiator used.
- Based on light transmittance data and SEM micrographs, acidic gels are likely composed of smaller particles, forming smaller aggregates and pores with less light scattering, while basic gels have larger particles forming larger aggregates and pores leading to more light scattering and opacity.
- UV/VIS spectroscopy was useful for indicating the gel point, marked by an inflection point in light transmittance over time. Differentiating light transmittance spectra using various orders of derivatives enhanced time resolution for precisely and accurately determining the gelation point.

## 4. Materials and Methods

### 4.1. Independent Variables

#### 4.1.1. pH

Our specific requirement is to develop a paste consisting of bioactive glass and WG that can be applied to fill bone defects and set within a practical timeframe for surgical procedures. Considering the time necessary to manipulate and shape the paste at the defect site before it solidifies significantly, and to ensure adequate time for sample preparation and characterization, we set a target gelation time of 10 min. The variable in our study is the pH at which these gels achieve the desired gelation time of 10 min. This variability in pH allows us to explore different formulations to ensure the gel remains effective and safe for clinical use. We limited the pH range to above 2 because pH levels below this threshold are generally considered unsafe or impractical for most applications.

#### 4.1.2. Type of Acid Initiator

We selected boric acid (BA) and phosphoric acid (PA) as acid initiators because both have been used in different biomaterials such as contact lenses [56], and dental and bone cements [57,58]. Moreover, this selection allows us to compare and contrast the effects of a relatively weaker acid (BA) and a stronger acid (PA) on gelation kinetics and final gel properties.

BA (H_3_BO_3_) acts as a monobasic acid in aqueous solutions. BA either consumes a strong base like a hydroxide ion, or reacts with water to release a proton [59]:

(10) $$B{\left(\mathrm{OH}\right)}_{3}+{\;H}_{2}O\;↔\;B{\left(\mathrm{OH}\right)}_{4\left(\mathrm{aq}\right)}^{-}+{\;H}_{3}O_{\left(\mathrm{aq}\right)}^{+}\;\mathrm{pka}=9.24$$

PA (H_3_PO_4_) is a polyprotic acid and its dissociation occurs in three steps depending on the pH [60]:

(11) $$H_{3}{\mathrm{PO}}_{4}+H_{2}O\;↔{\;H}_{2}{\mathrm{PO}}_{4\;\left(\mathrm{aq}\right)}^{-}+{\;H}_{3}O_{\left(\mathrm{aq}\right)}^{+}\;\mathrm{pk}\;=2.12$$

(12) $$H_{2}{\mathrm{PO}}_{4\;\left(\mathrm{aq}\right)}^{-}+{\;H}_{2}O\;↔{\;\mathrm{HPO}}_{4\;\left(\mathrm{aq}\right)}^{2-}+{\;H}_{3}O_{\left(\mathrm{aq}\right)}^{+}$$

(13) $${\mathrm{HPO}}_{4\;\left(\mathrm{aq}\right)}^{2-}+{\;H}_{2}O↔{\;\mathrm{PO}}_{4\;\left(\mathrm{aq}\right)}^{3-}+{\;H}_{3}O_{\left(\mathrm{aq}\right)}^{+}\;$$

#### 4.1.3. Waterglass Concentration

Waterglass concentration significantly affects its viscosity and tackiness, and consequently influences its properties as a binder. A key criterion to consider is that after mixing the binder with bioactive glass the paste should be formable and stable enough to fill a bone defect of any shape. Waterglass:water ratio of 1:1 was the minimum ratio at which the viscosity of the binder allowed homogenous mixing with acid solution. Conversely, a ratio of 1:5 was found to be the maximum at which the binder maintained enough adhesion to create a formable paste that retained its shape after demolding from a cylindrical mold.

### 4.2. Materials

The starting commercial sodium silicate solution (Sigma-Aldrich, CAS number: 6834-92-0), St. Louis, MO, U.S.A.) had the following characteristics: [SiO_2_] = 26.5 wt.%, [Si] = 6.13 mol/L; relative density = 1.39 g/L; pH = 11.9; SiO_2_/Na_2_O molar ratio = 2.57. The acid initiators used were phosphoric acid, 85% solution (Fisher Scientific, CAS number:7664-38-2, Toronto, ON, Canada) and boric acid powder, ≥99.5% (Fisher Scientific, CAS number: 10043-35-3, Toronto, ON, Canada).

### 4.3. Preparation of Dilute Waterglass and Acid Solutions

The dilute waterglass solutions were prepared by adding DIUF water to the commercial solution at room temperature under magnetic stirring to achieve waterglass to water weight ratios of 1:1, 1:3, and 1:5. To minimize the water introduced with the acid solution, a 0.7 M BA solution was prepared by dissolving 4.3 g of BA powder into 100 mL of DIUF water, which was near the maximum solubility of BA at room temperature. Additionally, a 14.8 M PA solution was prepared by mixing 11 g of 85% PA solution with 1 mL of DIUF water, achieving the maximum PA concentration with a sufficiently low viscosity for micropipette use. The compositions and pH values of the starting diluted waterglass and acid solutions are reported in Table 4.

### 4.4. Gelation Time

Gelation time, defined as the interval between acid introduction and the gel formation point, can be determined using qualitative, semi-quantitative, or quantitative methods. Qualitative approaches observe the point when the solution stops flowing upon tilting [18,25,50] or inversion [61,62], while semi-quantitative methods use predefined codes to describe the gelation process [63], subjective to observer judgment. Quantitative techniques, on the other hand, employ rheological measurements in static [17,64] or dynamic [31,65,66] modes. Due to its simplicity, speed, good agreement with qualitative methods [67], and occurrence at the formation of a completely rigid gel, we utilized the tube inversion method to measure the gelation time.

Four series of pH-adjusted samples were prepared by adding increasing amounts of acid initiator to 20 g of initial diluted waterglass solutions of varying concentrations (1:1, 1:3, and 1:5) under continuous stirring. The volume of acid in each sample was adjusted based on the waterglass concentration solution and the type of acid initiator:

PA specimens:

PA solution was added using a micropipette to achieve gelation times of less than 90 min, recorded as the minimum acid required in the basic region for each concentration. From the minimum acid amount, additional acid was added stepwise to subsequent samples of the same concentration. Specifically, 5 µL, 10 µL, and 20 µL of acid were added in each step for PA 1:1, PA 1:3, and PA 1:5, respectively. This process continued until the instant gelation zone in the basic region was reached. After this point, acid was added incrementally until the gelation time increased again, marking the minimum acid required in the acidic region. From this point, 100 µL of acid was added to the minimum amount in each step for all concentrations.

BA specimens:

BA solution was added using a 10 mL syringe to achieve gelation times of less than 90 min in the basic region. Then, 1 mL of acid was added at each step until reaching the instant gelation zone. The initial waterglass concentration of the solution for all BA specimens was 1:1 (total of one series of BA specimens) which was increased in each step (up to reaching rapid gelation) based on the amount of water introduced from the BA solution. No acidic gel was prepared using boric acid due to its weak acidic nature.

For both PA and BA specimens, the pH of each mixture was measured using a pH meter (Accumet Basic AB15 Plus, Fisher Scientific, Toronto, ON, Canada) with the pH probe inside the solutions and the final pH was recorded two minutes after acid addition. The pH meter was calibrated using buffer solutions on a daily basis. Based on the pH range being the measure, three buffer solutions among pHs of 2, 4, 7, 10, and 12 were used for calibration. The solutions were then transferred to 15 mL glass vials, filling up to ¾ of the total volume. The vials were gently inverted 180° (turned upside down) at regular time intervals, depending on the gelation rate, to observe the flow of the solution. The time at which the solution ceased to flow upon inversion was recorded as the gelation time.

### 4.5. Optical Properties

#### 4.5.1. Macrographs

Macrographs were used in this study for qualitative analysis of optical properties and to observe the Tyndall light scattering effect during, and after, the sol–gel transition. All macrographs were obtained using a digital single-lens reflex camera (D300s, Nikon, Nikon Corporation, Minato City, Tokyo, Japan) equipped with an AF Micro-Nikkor 60 mm f/2.8D lens (Nikon Corporation, Minato City, Tokyo, Japan). A setup consisting of a stand holding a black non-reflective velvet fabric as the background was used for capturing photos. The camera exposure settings were kept constant for all photos. Only specimens with target gelation time of 10 min were photographed.

##### Sol–Gel Transition

Fresh mixtures of waterglass solutions and acid solutions were prepared, poured into 10 mL glass vials, and placed on the non-reflective stage. Photos were taken at 2 min intervals during the sol–gel transition, at the gelation point, and at 5, 15, 30, 45, and 60 min after gelation. Before reaching the gelation point, the samples were leaned against a metal piece at a 45-degree angle to show that they still had flow. After the gelation point, the samples were photographed in an inverted position.

##### Tyndall Effect

One of the characteristic properties of disperse, or colloidal, systems is the Tyndall effect (or Faraday–Tyndall effect), which is the ability of these systems to scatter light in all directions when illuminated from one side with visible light. A colloidal dispersion, or colloid, is a suspension of tiny particles in some liquid medium. These suspended particles are single large molecules, or aggregates of molecules, or ions ranging in size from 1 to 1000 nanometers. Although undetectable in normal lighting, the presence of suspended particles can be demonstrated by shining a beam of intense collimated light—typically a laser beam—through the suspension. The beam is visible from the side because the light is scattered by the suspended particles. In a true solution, on the other hand, the beam is invisible from the side because the individual ions and molecules dispersed in the solution are too small to scatter visible light.

To observe the Tyndall effect, a set of specimens with gelation time of 10 min was freshly prepared, poured into 10 mL glass vials, and placed on the non-reflective stage. The vials were then illuminated using a green 550 nm laser pointer to observe the Tyndall effect and light scattering in the gels. The laser setup was prepared with the laser beam aligned both horizontally and vertically to pass through the central plane of the sample. Photos were taken in a dark room with the laser being turned on to illuminate the samples at 2 min intervals during the sol–gel transition, at the gelation point, and at 5, 15, 30, 45, and 60 min after gelation.

##### 4.5.2. UV/VIS Spectrophotometry

A UV/VIS spectrophotometer (Hitachi U-3900H, Hitachi High-Tech Corporation, Tokyo, Japan) was used to measure light transmittance in the gels. First, an empty cuvette was placed in the spectrophotometer, which was run from 400 to 700 nm for baseline correction. Fresh mixtures of waterglass and acid solutions were then prepared, poured into the cuvette, and placed in the spectrophotometer. Transmittance (T; in %) was measured at 2 min intervals during the sol–gel transition, at the gelation point, and at 5, 15, 30, 45, and 60 min after gelation.

The numerical values obtained in UV/VIS experiments can be presented as Transmittance or Absorbance plotted against wavelength. Transmittance (*T*), representing the light passing through a sample, is typically expressed as a fraction of the transmitted radiation (*I*) over the incident radiation (*I*_0_) and is defined as follows:

(14) $$T=\frac{I}{I_{0}}\;$$

Using Lambert–Beer’s law, “absorbance” (*A*) is calculated as follows:

(15) $$A=-\log T=\log\frac{I_{0}}{I}=\varepsilon cd$$

where *ε* is molar absorption coefficient; *c* is concentration; and *d* is the path length of the measuring beam in the sample.

While absorbance values are commonly used due to their linear relationship with concentration and path length, light interacts with samples in various ways beyond absorption, including reflection, scattering, and transmission. The terms “absorption” and “extinction” are often used interchangeably, which can be misleading in colloidal solutions and gels. “Absorption” refers specifically to the reduction of light intensity due to molecular excitation, whereas “extinction” includes absorption as well as scattering and reflection. As Mantele and Deniz [68] note, extinction accounts for all light loss, including scattering, which is significant in sol–gel transitions like waterglass. Thus, UV/VIS results are reported as T (%) with the remainder being extinction, mainly due to scattering.

### 4.6. Scanning Electron Microscopy

Scanning electron microscopy (SEM) was employed to image and compare the packing, porosity, and morphology of gels with a target gelation time of 10 min. The goal was to find correlations between the optical properties and microstructure of the gels prepared with different processing conditions.

To prepare the samples, fresh mixtures of waterglass solutions of different concentrations and acids were made and poured into 1 × 1 × 1 cm^3^ silicon molds, where they were allowed to gel. Then, 60 min after the gelation point, the samples were immersed in liquid nitrogen to freeze. After freezing, the samples were dried using a Savant SuperModulyo Freeze Dryer (Mechatech System Ltd., Thornbury, Bristol, UK) at a temperature of −40 °C and a pressure of ~0.08 Torr for 12 h. Prior to imaging, the specimens were sputtered with gold using Denton Desk II (Denton Vacuum, Moorestown, NJ, USA) for 90 s, resulting in a 12 nm gold coating to reduce charging. Imaging was conducted in Secondary Electron (SE) mode with a Zeiss EVO MA10 SEM (Carl Zeiss AG, Oberkochen, Baden-Württemberg, Germany) equipped with a LaB_6_ filament.

## Figures and Tables

**Table 1:** The equations fitted to gelation time (t_g_) versus pH curves obtained for basic gels made with boric acid, and basic and acidic gels made with phosphoric acid.

Acid Catalyst	WG:Water (wt. Ratio)		WG (wt%)	Fitted Equation	Parameters			
					a	b	c	d
**Boric Acid**	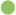 1:5	Basic	15	t_g_ = a·e^b·pH^	1 × 10^−27^	6.107	-	-
**Phosphoric Acid**	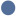 1:1	Basic	50		2 × 10^−60^	13.135	-	-
	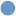 1:3		25		5 × 10^−40^	8.929		
	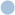 1:5		15		5 × 10^−26^	6.221		
	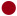 1:1	Acidic	50	t_g_ = a·pH^3^ + b·pH^2^ + c·pH + d	−5 × 10^−5^	0.002	−0.114	100.47
	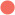 1:3		25		−5 × 10^−5^	0.004	−0.134	100.74
	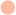 1:5		15		−6 × 10^−5^	0.011	−0.823	100.38

**Table 2:** This graph illustrates the composition and final pH of silica gels made with boric acid and phosphoric acid at different waterglass concentrations. All gels have a gelation time of ten minutes, making them practical for clinical applications. The seven specimens shown are the primary samples in this study, used to investigate the effects of pH, waterglass concentration, and the type of acid initiator.

Acid Catalyst	WG:Water (wt. Ratio)		WG (g)	Acid Content (µL)	pH	t_g_ (min)
**Boric Acid**	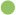 1:5	Basic	3.34	14,000	10.60	10
**Phosphoric** **Acid**	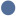 1:1	Basic	10.00	545	10.63	10
	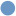 1:3		5.00	360	10.35	
	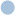 1:5		3.34	300	9.70	
	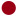 1:1	Acidic	10.00	2800	3.36	10
	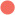 1:3		5.00	1300	4.80	
	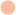 1:5		3.34	825	5.30	

**Table 3:** The parameters of the third-order polynomials fitted to the light transmittance data of different silica gels.

Acid Catalyst	WG:Water (wt. Ratio)		WG (wt%)	Fitted Equation	Parameters			
					a	b	c	d
**Boric Acid**	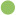 1:5	Basic	15	%T = at^3^ + bt^2^ + ct + d	25 × 10^−6^	0.321	−9.155	119.7
**Phosphoric Acid**	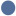 1:1	Basic	50		−23 × 10^−5^	0.176	0.193	99.92
	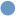 1:3		25		−49 × 10^−5^	0.081	−4.498	114.5
	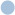 1:5		15		−26 × 10^−6^	0.041	−2.428	107.7
	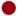 1:1	Acidic	50	%T = at^3^ + bt^2^ + ct + d	−19 × 10^−6^	0.002	0.114	100.5
	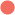 1:3		25		51 × 10^−6^	0.004	0.134	100.7
	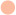 1:5		15		56 × 10^−6^	0.10	−0.823	100.4

**Table 4:** Compositions and pH values of the prepared starting waterglass and acid solutions.

	WG:Water (wt. Ratio)	Water (g)	WG (g)	pH		Acid Type	Acid: Water (wt. Ratio)	Water (g)	Acid (g)	pH
**WG** **Solutions**	1:1	10.00	10.00	11.48	**Acid Solutions**	PA	11:1	1.00	11.00	1.50
	1:3	15.00	5.00	11.22		BA	1:24	96.00	4.00	3.20
	1:5	16.66	3.34	10.91						

**Figure A1:**
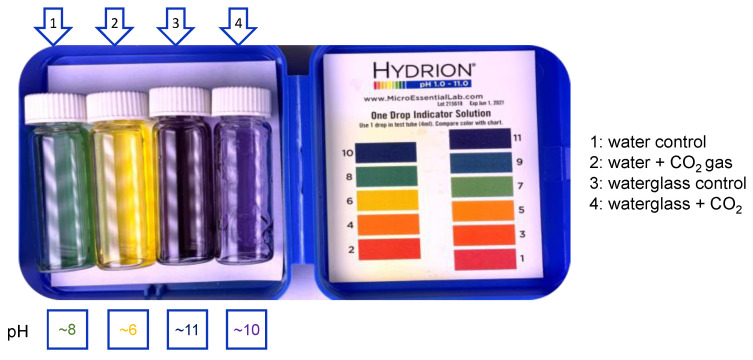
Water and waterglass solutions before and after CO_2_ gassing using a bicycle CO_2_ inflator. Each solution contains pH indicator, with the color change representing the decrease in pH due to the introduction of CO_2_.

**Figure A2:**
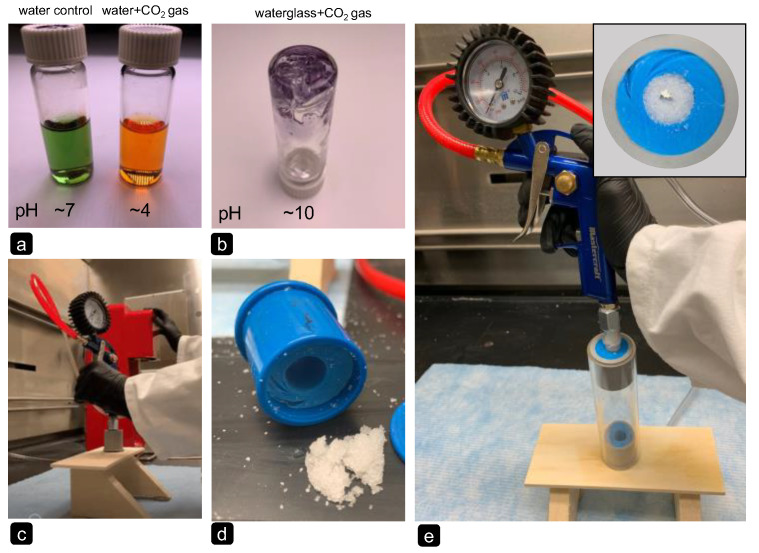
(**a**) Water solutions containing pH indicator before and after CO_2_ gassing using a Soda Stream machine. (**b**) Waterglass containing pH indicator after CO_2_ gassing using a Soda Stream machine, with a layer of gel formed after gassing. (**c**) The procedure of CO_2_ gassing of waterglass–bioactive glass paste inside an open cylindrical mold using a Soda Stream machine. (**d**) Damage to the waterglass–bioactive glass paste due to high concentrated pressure. (**e**) We used a long plastic cylinder to increase the distance of the nozzle. However, some specimens were still damaged by the concentrated pressure, as shown in the top right.

**Figure A3:**
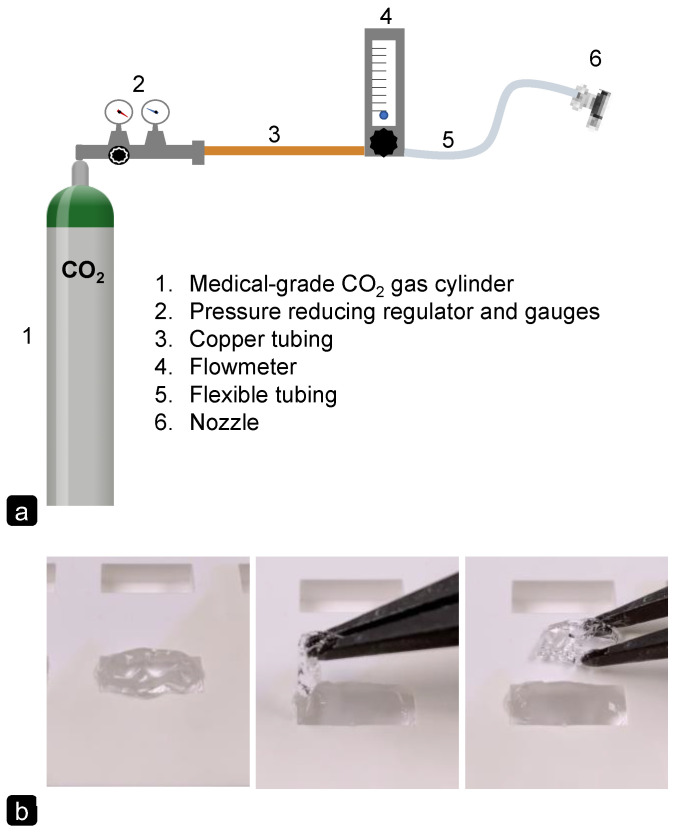
(**a**) Illustration detailing the components used to assemble a medical-grade concentrated CO_2_ gas system. (**b**) Image showing a negative silicone mold filled with waterglass, highlighting the formation of a thin silica gel film after exposure to medical-grade CO_2_.

**Figure A4:**
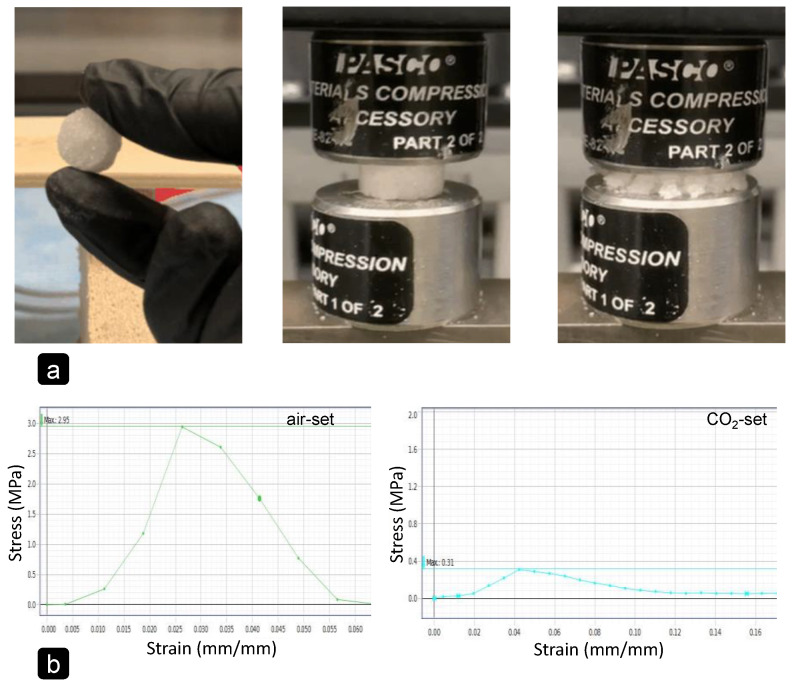
(**a**) The image on the left shows the composite holding its shape after waterglass gelation. The two images on the right demonstrate how the composite deforms under compressive load. (**b**) These curves compare the mechanical properties of air-set and CO_2_-set waterglass–bioactive glass composites.

## Data Availability

The raw data supporting the conclusions of this article will be made available by the authors upon request.

## Appendix A. Why Pure CO_2_ Gas Did Not Work

Carbon dioxide gas is widely used to harden the sodium silicate-bonded sand systems in the metal casting industry. The so-called “CO_2_ process” was first conceptualized in 1898 and has gained a lot of acceptance since the 1950s. The process is based on forming a bond by setting the waterglass through brief exposure to CO_2_ gas to bind sand grains together. The following chemical equation was suggested for representing the reaction between carbon dioxide and water glass:

(A1) $${\mathrm{Na}}_{2}O\cdot {\mathrm{xSiO}}_{2}\cdot {\mathrm{yH}}_{2}O\;+{\mathrm{CO}}_{2\;}\to {\;\mathrm{Na}}_{2}{\mathrm{CO}}_{3\;}+{\mathrm{xSiO}}_{2}\cdot {\mathrm{yH}}_{2}O\;$$

This equation is believed to be over-simplified [69]. Nonetheless, it has been generally accepted that the bonding between the sand grains in the carbon dioxide process is due to silica gel formation.

Based on the results of previous proof-of-concept work, it was hypothesised that the setting time of waterglass–bioglass composites could be accelerated by using concentrated CO_2_ gas, instead of letting them air-set with atmospheric CO_2_. To put this hypothesis to the test, a series of preliminary experiments were performed, as detailed below.

First, a bicycle tire CO_2_ inflator was used to measure the effect of introducing CO_2_ on lowering pH in aqueous environments. The tip of the inflator was inserted into glass vials containing water and waterglass solutions mixed with a pH indicator solution, and gas was introduced until no further change in the color of the solution was observed. The results are shown in Figure A1, where the pH of the solutions can be compared before and after the introduction of CO_2_ gas. As seen, the pH was lowered in both the water and waterglass solutions due to the formation of carbonic acid. However, the lowest pH achieved in waterglass was approximately pH = 10.

Next, a Soda Stream machine (SodaStream, London, UK) was used to apply concentrated CO_2_ gas to a mixture of waterglass and 45S5 bioactive glass paste. With its higher pressure, the soda stream machine reduced the pH of the water to approximately 4 (see Figure A2a), which is lower compared to the tire inflator. However, with waterglass, the pH remained around 10. This lack of significant change compared to the CO_2_ tire inflator could be due to carbonic acid being a weak acid and the buffering ability of waterglass. Although the formation of silica gel after about 15 min was confirmed using the tube inversion method (Figure A2b), the gel was only formed on the surface and not throughout the entire bulk, stopping the flow of the entire solution. This skin effect was evident when stirring the solution with a glass rod; it reverted to a solution state.

After mixing bioactive glass and waterglass (according to the optimal ratios in Guzzo’s work [8,9]), the paste was inserted into an open-ended cylindrical mold. The CO_2_ gas was guided to the surface of the paste using a plastic tube and a nozzle with a pressure gauge (see Figure A2c). The pressure fluctuated between 2 and 20 psi and in some cases, the concentrated pressure was so high that it destroyed the entire specimen (see Figure A2d). To solve this problem, a long plastic cylindrical tube with the same width as the mold was used on top of the mold to increase the distance from the tip of the nozzle. However, the problem of having no control over the pressure and flow persisted, and in some cases, the specimens were pierced due to the high pressure (see Figure A2e).

For better control over the gas pressure and flow, a CO_2_ gas setup was used, consisting of a medical-grade CO_2_ gas cylinder (Praxair, Danbury, CT, USA), a pressure regulator (Harris Products Group, Mason, OH, U.S.A.), LLDPE tubing (0.25” by 0.17”, max 140 psi at 70 °F), a flowmeter (Cole-Parmer, Vernon Hills, IL, U.S.A.), and a nozzle (Figure A3a). Waterglass was poured into silicone rubber molds measuring 0.4 × 0.4 × 1.0 cm. To initiate gelation, a gas stream was introduced by positioning the nozzle as close as possible to the surface of the solution and moving it side to side to ensure even coverage. The gas flow rate was maintained below 5 L/min to prevent splashing. A thin gel film was observed forming on the surface, which could be easily removed with tweezers (see Figure A3b). Despite five minutes of gassing, only this thin film of gel was observed, with no gelation occurring in the bulk of the solution. This behavior can be attributed to mass transport limitations, where the rate of gelation at the surface outpaces the diffusion of reactants (CO_2_ gas) into the bulk solution, thereby inhibiting the formation of a gel network throughout the entire volume.

At this point, it was hypothesized that since the bioactive glass particles would be coated with thin layers of waterglass binder after mixing, forming a thin layer of gel might be sufficient to set the entire composite. Therefore, the mixture of bioactive glass and waterglass was poured into cylindrical molds and gassed using the CO_2_ setup. The gassed composites were then tested in compression immediately after gassing from both sides using a PASCO materials testing tabletop machine equipped with a 7100 N load cell (PASCO Scientific Inc., Roseville, CA, USA).

The strength of the composite compact is influenced by factors such as the SiO_2_ ratio of the sodium silicate, gassing time, flow rate, and the water content of the silica gel [70]. We explored various gassing times (30–360 s) and flow rates (5, 7, and 10 L/min) at a constant pressure of 5 psi. Although the final com.posites retained their shape and integrity after gassing (see Figure A4a), the mechanical testing results were generally inconsistent, and no meaningful correlation was found between compressive strength and the gassing variables. An example of the compressive stress-strain curves for air-set and CO_2_-set composites is shown in Figure A4b. Generally, the lower strength of CO_2_-set composites contrasted to the air-set ones was expected, as the air-set composites had completely dried over a 10-day period, whereas the CO_2_-set composites remained in a wet state.

Despite successfully setting the composite within minutes, several major drawbacks were encountered with this method, which are detailed as follows:

1. Mass transport limitations: The setting reaction is mass-transport limited, meaning that it is limited by diffusion of CO_2_ gas into waterglass. As gasification progresses, the surface viscosity increases to a point where it hinders further reaction, often leaving the reaction incomplete.
2. Ambiguities in Reaction Kinetics and Setting Point: Monitoring or controlling the setting point or the degree of gelation within the bulk is challenging. Chemical analysis techniques like SEM/EDX, XRD, Raman, and FTIR spectroscopy are not very useful due to the common elements and chemical bonds between sodium silicate gel and bioactive glass, as well as their amorphous nature. Physical analysis methods such as Micro-CT or light microscopy are also limited due to the similar densities and the transparent nature of the components.
3. Risk of under-gassing/over-gassing: Finding the optimal gassing conditions for ideal gelation kinetics is crucial. Insufficient gassing can result in incomplete reaction, while excessive gassing—known as “over-gassing”—can significantly reduce binder strength. Over-gassing occurs due to gel dehydration, accelerated evaporation, hydration of sodium carbonate crystals, or formation of sodium bicarbonate [69]. Since the reaction kinetics cannot be easily monitored, both of these cases are likely to happen.
4. Limited pH Range: The pH of waterglass is a key factor in controlling gelation kinetics and final gel properties. Ideally, a wide pH range should be explored to find the best processing conditions for our application and target gelation time. However, the carbonic acid introduced from CO_2_ gas is a weak acid and, combined with the buffering ability of the waterglass solution, cannot lower the pH below ~10. This limitation is problematic because the minimum gelation time in silica–water systems typically occur around neutral pH, which is also ideal for biological applications to reduce tissue damage.
5. Lack of Repeatability: No meaningful correlation was found between gassing variables (such as time and flow rate) and the final compressive strength. Other factors, including the sample’s height, the binder-to-bioactive glass ratio, the type of nozzle, and binder viscosity, also influence the results. With so many variables and no control or clear understanding of the gelation progression, achieving consistent results became nearly impossible.

Therefore, due to these limitations, we decided to find solutions instead of use CO_2_ gas to induce gelation in waterglass.
